# The identification of liver cirrhosis with modified LBP grayscaling and Otsu binarization

**DOI:** 10.1186/s40064-016-1970-6

**Published:** 2016-03-12

**Authors:** Karan Aggarwal, Manjit Singh Bhamrah, Hardeep Singh Ryait

**Affiliations:** Department of Electronics and Communication Engineering, Punjabi University, Patiala, India; Electronics and Communication Engineering Department, BBSBC, Fatehgarh Sahib, India

**Keywords:** Cirrhotic liver, Ultrasound image, Differential local binary pattern

## Abstract

Liver cirrhosis is considered as one of the most common diseases in healthcare. The widely accepted technology for the diagnosis of liver cirrhosis is via ultrasound imaging. This paper presents a technique for detecting the cirrhosis of liver through ultrasound images. The region of interest has been selected from these ultrasound images and endorsed from a radiologist. The identification of liver cirrhosis is finally detected through modified local binary pattern and OTSU methods. Experimental results from the proposed method demonstrated its feasibility and applicability for high performance cirrhotic liver identification.

## Background

World Health Organization (WHO) states that 130–150 million people globally have chronic hepatitis C infection. Lot many those patients may develop liver cirrhosis due to poor medical attention (WHO 2015). Cirrhosis is considered to be the end stage of chronic hepatopathies which may leads to hepatocellular carcinoma (Virmani et al. 2013). The diagnosis of the liver cirrhosis is best achieved by looking at the granular structure of the liver parenchyma and the aspects of the liver surface such as its unevenness and roughness (Virmani et al. 2011). Ultrasound imaging techniques are widely used to diagnose liver cirrhosis using the advanced image processing tools and techniques.

**Fig. 1:**
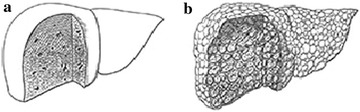
**a** Normal liver, **b** cirrhosis liver: provided by National Institute of Diabetes and Digestive and Kidney Diseases, National Institutes of Health (NIDDK 2010)

In the present medical scenario, a physician’s experience matters to diagnose the cirrhosis where often it is felt that assistive technologies must be developed to improve ultrasound images. Therefore, researchers have been actively exploring the quantitative method to characterize and analyze the different stages of cirrhotic liver (Fujino et al. 2014). Biopsy is considered as “golden standard” for diagnosing diffuse liver diseases, but being in vivo in nature usually not easily accepted the patient. Ultrasonography is the most preferred examination for screening, mainly due to its noninvasive and nonradioactive nature (Jeong et al. 2007). Here, it has been established by the radiologist that the texture of the body tissues provides an important visual property in classification and characterization of results in their radiological significant (Mitrea et al. 2012). Image texture can be qualitatively defined in following categories of fineness, coarseness, smoothness, surface granulation, randomness, irregular or hummocky (Hawlick 1979). In the textural images, the intensity and spatial arrangement of pixels describes all the above mentioned features (Castellano et al. 2004). This irregular pattern/hummocky is due to rough as well as eruptions/sores/scares on the surface of cirrhotic liver. The regular pattern in normal liver and irregular pattern in cirrhotic liver is shown in Fig. 1a.

Image texture can be qualitatively defined in following categories of fineness, coarseness, smoothness, surface granulation, randomness, irregular or hummocky. In the textural images, the intensity and spatial arrangement of pixels describes all the above mentioned features.

The most common pattern or hummocky of human liver disease is micronodular cirrhosis, a condition characterized by fibrosis and the conversion of normal liver architecture into uniform sized regenerating small nodules. Microscopically, normal liver architecture is distorted by scar tissue which forms a band of connecting tissue joining the periportal and perivenous areas. It is often said that micronodular cirrhosis may be converted into cirrhosis of macronodular type (Fauerholdt et al. 1983). The progression may lead to complete cirrhosis. This becomes a very tedious job to differentiate the change when ultrasound scanning is done. Radiologist needs a very critical analysis to differentiate between these two. If a diagnostic tool can be developed based on the concept of image processing, early disease detection is possible.

Of course, ultrasound images are to be taken for spatial domain analysis as texture differentiation appears to be the vital parameter (Lee 2013; Wu et al. 2012, 2013). Here, the main problem lies in identifying smaller granular or hummocky bodies (about the size of millet seed measuring from 1 to 2.5 mm in diameter) in case of normal liver and substantially, clustered and inflammatted matrices of granular hummocky bodies (because of scarring) for the case of Cirrhotic Liver. The widely accepted techniques in spatial domain are LBP and OTSU methods. LBP technique reduces a grayscale image to mean weighted grayscale image i.e. grayscale value of central pixel is calculated as per the mean of its surrounding pixels which is defined by the radius parameter of LBP technique.

Regeneration of the ultrasound image on the basis of local binary patterns (LBP) technique has been applied to various uniform texture patterns detection (Brunenberg et al. 2006; Caballero et al. 2006; Keramidas et al. 2007; Pujol et al. 2003). The most important properties of LBP are its tolerance regarding monotonic illumination changes and its computational simplicity. LBP was originally proposed for texture analysis (Ojala et al. 1996) and hence proved to be a simple yet powerful approach to describe local structures. It has been extensively exploited in many applications, for instance, face image analysis (Ahonen et al. 2004; Hadid et al. 2004), image and video retrieval (Huijsmans and Sebe 2003; Grangier and Bengio 2008), environment modeling (Ali et al. 2008; Nanni and Lumini 2008), visual inspection (Maenpaa et al. 2003; Turtinen et al. 2006), motion analysis (Heikkila and Pietikainen 2006; Kellokumpu et al. 2008), biomedical and aerial image analysis (Oliver et al. 2007; Kluckner et al. 2007), and remote sensing (Lucieer et al. 2005). However, this very technique can be seen very useful for cirrhotic liver images obtained through ultrasound.

On the other hand, Otsu’s method (Rodríguez 2006; Huang and Wang 2009) a high-speed and effective thresholding approach is applied for image binarization. In this method, it is mainly exploited to discriminate the background and objects on a gray level histogram. It has indeed the advantage to be highly efficient and demands less computation time when the number of classes is two (Tamim et al. 2015; Filipczuk et al. 2013).

The present research work is an effort to detect and classify liver cirrhosis based on their structure and texture pattern retrieved from USI. During the past few years, LBP has aroused increasing interest in image processing and computer vision due to its nature of nonparametric method and Otsu method has the capability in changing grayscale images to binary images using adaptive thresholding. That’s why OTSU is additionally added to LBP generated weighted gray scale image. OTSU is doing its normal job but it is applied not to the original image but to the modified LBP image. This modification of LBP method is represented as differential local binary pattern (DLBP) in our further discussion.

## Methodology

**Fig. 2:**
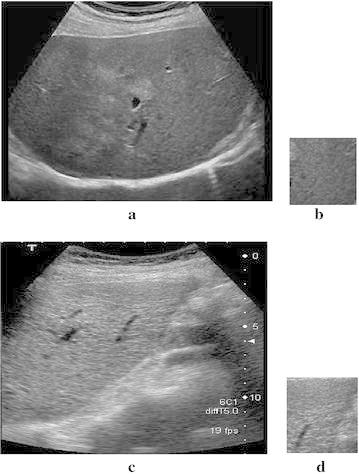
**a** Ultrasound image of normal liver, **b** ROI defined by radiologist from normal liver, **c** ultrasound image of cirrhosis liver, **d** ROI defined by radiologist from cirrhosis liver

The US images were acquired from radiology center under supervision of an experienced radiologist. The ultrasound images each of size 381 by 331 in JPG format were specified in two categories as normal and cirrhotic liver. All the images were acquired from same ultrasound machine to nullify the effects of change in dimensions and texture values on the images which may have happened if it is taken by different sources. The ultrasound probe used for acquiring these images was having depth of 15 cm and frequency of 5 MHz. Radiologist also specified the portion in the images which belonged to cirrhosis as shown in Fig. 2 for cross evaluation. Region of interest (ROI) has been selected as templates of size 50 × 50 each from both normal liver and cirrhotic liver ultrasound images as in Fig. 2a–d.

Accordingly, one template was selected from each image, i.e. total of 6 templates were taken from 6 images of normal liver. Similarly, 44 templates were selected from 44 images of cirrhotic liver. Modified LBP with OTSU approach was applied on to the selected templates of both normal and cirrhotic liver.

**Fig. 3:**
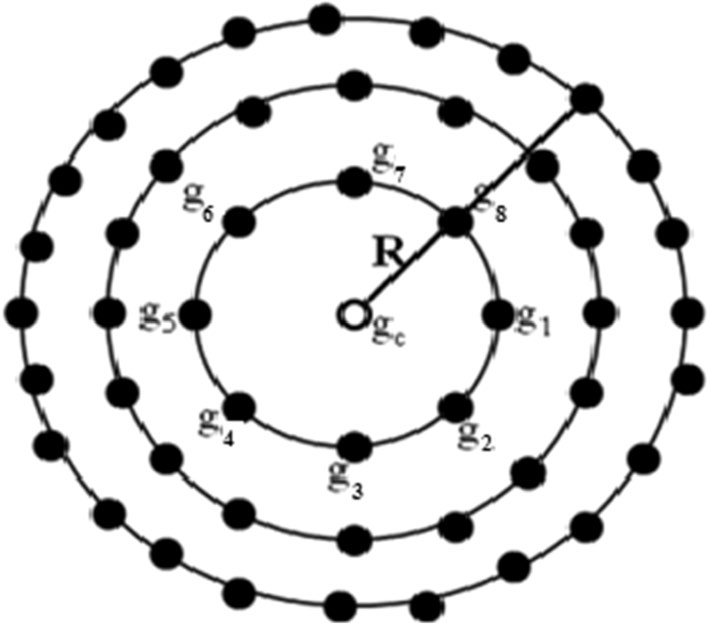
Circular neighborhood set for different (P, R)

Ojala et al. (2002) presented the LBP operator for texture classification. LBP operator is that value of the pixel where the accumulative value is calculated from comparing its gray value with its neighbors as given in Eq. (1).

1 $$LBP_{P,R} \left( {x_{c} ,y_{c} } \right) = \mathop \sum \limits_{p = 1}^{P} s\left( {g_{p} - g_{c} } \right)2^{P - 1}$$

where

2 $$s\left( z \right) = \left\{ {\begin{array}{*{20}c} {1, \quad z \ge 0} \\ {0, \quad z < 0} \\ \end{array} } \right.$$

where *g*_*c*_ is the gray value of the center pixel, *g*_*p*_ is the gray value of neighbor at radius *R* from the center pixel (*g*_*c*_) and *P* is the number of neighbors at a distance (radius) *R* from the center pixel (*g*_*c*_) in an image. Examples of such circular neighbor sets for different configurations of (*P*, *R*) are shown in Fig. 3. The LBP operator when processed onto the entire image creates new LBP image with desired intensity at each pixels. The concept used to get this desired intensity at pixel level is:

If the gray level of the neighboring pixel is higher or equal to the center pixel, the value is set to one otherwise the value is zero.

Otsu’s method is a clustering-based image thresholding, which is based on the selection of the optimal thresholds from the gray-level histogram of an image that maximizes the between-class variance (Fukunage 1972). Otsu defines the between-class variance using the discriminate analysis based on statistics of the 1-D histogram (Otsu 1979).

Assuming an image with the size M × N and gray range L, the number of pixels at gray level i is denoted by n_i_, thus the probability of gray level i can be gained by

$${\text{P}}_{\text{i}} = {\text{n}}_{\text{i}} /\left( {{\text{M}} \times {\text{N}}} \right),\quad \quad {\text{P}}_{\text{i}} \ge 0;\quad \mathop \sum \limits_{0}^{{{\text{L}} - 1}} {\text{P}}_{\text{i}} = 1$$

If we divide the pixels of the image into two classes of object C_0_ and background C_1_ by the threshold t, the gray level probability distributions for the two classes can be expressed by

$$\upomega_{0} = {\text{P}}_{\text{r}} \left( {{\text{C}}_{0} } \right) = \mathop \sum \limits_{{{\text{i}} = 0}}^{\text{t}} {\text{P}}_{\text{i}} =\upomega\left( {\text{t}} \right)$$

$$\upomega_{1} = {\text{P}}_{\text{r}} \left( {{\text{C}}_{1} } \right) = \mathop \sum \limits_{{{\text{i}} = {\text{t}} + 1}}^{{{\text{L}} - 1}} {\text{P}}_{\text{i}} = 1 -\upomega\left( {\text{t}} \right)$$

The gray means of the class C_0_ and C_1_ are

$$\upmu_{0} = \mathop \sum \limits_{{{\text{i}} = 0}}^{\text{t}} {\text{iP}}_{\text{r}} /\upomega_{0} =\upmu\left( {\text{t}} \right)/\upomega\left( {\text{t}} \right)$$

$$\upmu_{1} = \mathop \sum \limits_{{{\text{i}} = {\text{t}} + 1}}^{{{\text{L}} - 1}} {\text{iP}}_{\text{r}} /\upomega_{1} = \frac{{\upmu_{\text{T}} -\upmu\left( {\text{t}} \right)}}{{1 -\upomega\left( {\text{t}} \right)}}$$

where $$\upomega\left( {\text{t}} \right) = \sum\nolimits_{{{\text{i}} = 0}}^{\text{t}} {{\text{P}}_{\text{i}} }$$, $$\upmu\left( {\text{t}} \right) = \sum\nolimits_{{{\text{i}} = 0}}^{\text{t}} {{\text{iP}}_{\text{i}} }$$, $$\upmu_{\text{T}} = \sum\nolimits_{{{\text{i}} = 0}}^{{{\text{L}} - 1}} {{\text{iP}}_{\text{i}} }$$.

The between-cluster variance of the class C_0_ and C_1_ can be given by

$$\upsigma_{\text{B}}^{2} \left( {{\text{t}}^{*} } \right) =\upomega_{0} \left( {\upmu_{0} -\upmu_{\text{T}} } \right)^{2} +\upomega_{1} \left( {\upmu_{1} -\upmu_{\text{T}} } \right)^{2}$$

During the image segmentation process by the OTSU method, the between-cluster variance is considered as an important index for the uniformity of gray distributions (Chaou et al. 2015). The larger the between class variance is, the greater the difference between the two classes becomes. The optimal threshold ${\text{t}}^{*}$ can be figured out through maximizing the between-class variance *σ*_*B*_.

$$\sigma_{B}^{2} \left( {t^{*} } \right) = \mathop \sum \limits_{{{\text{t}} = 0}}^{{{\text{L}} - 1}} Max\left\{ {\sigma_{B}^{2} \left( t \right)} \right\}$$

Being a nonparametric approach, LBP summarizes local structures of images efficiently by comparing each pixel with its neighboring pixels (Murala and Jonathan 2014; Hu and Zhao 2010). Here, it is important to mark the radius i.e. *R* for the computation of LBP operator.

In the present study, a technique for detecting the liver cirrhosis through ultrasound images has been developed by exploiting LBP operator’s computing principle. Widely used local binary pattern encodes the relationship among the surrounding neighbors for a given referenced pixel in an image. The possible relationships among the surrounding neighbors are depending on the number of neighbors. The LBP approach has been observed as a good approach for texture analysis of regular pattern. Hence, found to be advantageous for simultaneously classification between a regular and an irregular pattern.

LBP technique has been slightly modified in terms of difference value between neighboring pixels and centre pixel. The modified approach is:

If the difference value is less than or equal to standard deviation of that matrix then difference value is replace by 0 and otherwise the difference value is replace by one.

Hence, the macro granular in the hummocky type structure of liver appears in the form of black portion in the binary images. Therefore, a technique is needed so that binarization and thresholding becomes so accurate to figure out difference between the normal and cirrhotic liver.

Now if the dimension or size of these black portions can be calculated in terms of no. of consecutive pixels either horizontally or vertically placed, classify between a normal texture and cirrhotic texture is possible.

In the present work the ideology used is to measure black portion or spot or granular as count of horizontally connected black pixels. This count is called here as connectivity. The concept is further extended by counting three such consecutive pixels then four and lastly five such pixels. These 3, 4 and 5 connected pixels can further be explored to segregate the stages of cirrhosis. The concept of radius is used to generate spatially equalized LBP grayscale image. Three such selection were made; firstly LBP with radius = 1, secondly = 2 and lastly = 3. Bigger the radius leads to more spatial equalization and thus less chance to detect irregularisation or hummocky type structure.

**Fig. 4:**
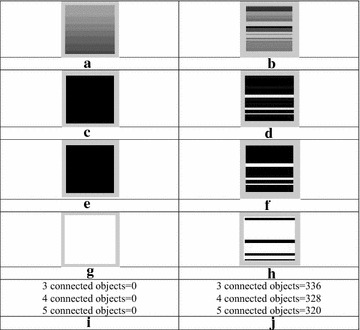
**a**, **b** Regular and irregular texture image, **c**, **d** LBPs’ images of regular and irregular images, **e**, **f** OTSU images i.e. results after applying OTSU on both LBPs’ images (**g**, **h**) complement of OTSU images (**i**, **j**) values of connected objects for both regular and irregular images

The same has been projected as an experiment below. In this, Fig. 4a, b were regular and irregular texture images. Figure 4c, d were LBPs’ images of regular and irregular images respectively. Figure 4e, f were the results after applying OTSU on LBPs’ images. Figure 4g, h were complement of the OTSU images.

If the image is of any regular texture as shown in Fig. 4a then regularity is in its gray level distribution. As first step, Modified LBP approach was applied which removed variation of grayscale and generated Fig. 4e. As there was no abrupt change of grayscale, compliment of 4e becomes a white (blank) image. Therefore, connectivity of black portion comes zero as shown in 4i. Similarly, if any image contains an irregular texture as in Fig. 4b, this means that there is a non-uniform distribution of its grayscale; therefore connecting of the cells will be increased as shown in Fig. 4h. There is a values in 3, 4 as well as in 5 connected pixels as shown the value in Fig. 4j.

**Fig. 5:**
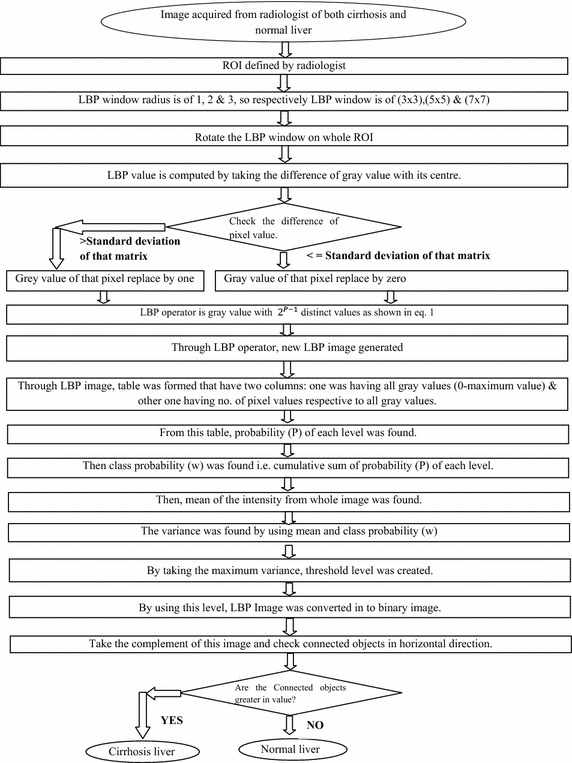
Flow chart of proposed method

In normal liver, because granular or hummocky structure is about uniform in sizes, the value will come very lesser in all 3, 4 or 5 connectivity. If cirrhotic liver is taken, due to the non uniformity, it may appear more. That is the basic of this connectivity. The detailed methodology has been presented in the flow chart shown in Fig. 5.

## Results and discussion

Firstly, by applying Otsu method directly to the all templates and get these all 3, 4 and 5 connected counts as shown in Table 1. This method is not giving any specific result.

**Table 1:** 3, 4 and 5 connected counts using Otsu method

Sr. No.	Liver	3 connected black objects	4 connected black objects	5 connected black objects
1	Cirrhosis	0	0	0
2	Cirrhosis	8	2	0
3	Cirrhosis	0	0	0
4	Cirrhosis	65	46	35
5	Cirrhosis	0	0	0
6	Cirrhosis	0	0	0
7	Cirrhosis	0	0	0
8	Cirrhosis	0	0	0
9	Cirrhosis	3	2	1
10	Cirrhosis	12	10	8
11	Cirrhosis	0	0	0
12	Cirrhosis	0	0	0
13	Cirrhosis	0	0	0
14	Cirrhosis	0	0	0
15	Cirrhosis	0	0	0
16	Cirrhosis	0	0	0
17	Cirrhosis	0	0	0
18	Cirrhosis	0	0	0
19	Cirrhosis	0	0	0
20	Cirrhosis	0	0	0
21	Cirrhosis	0	0	0
22	Cirrhosis	0	0	0
23	Cirrhosis	0	0	0
24	Cirrhosis	0	0	0
25	Cirrhosis	0	0	0
26	Cirrhosis	0	0	0
27	Cirrhosis	0	0	0
28	Cirrhosis	0	0	0
29	Cirrhosis	0	0	0
30	Cirrhosis	0	0	0
31	Cirrhosis	1	0	0
32	Cirrhosis	0	0	0
33	Cirrhosis	0	0	0
34	Cirrhosis	0	0	0
35	Cirrhosis	0	0	0
36	Cirrhosis	0	0	0
37	Cirrhosis	0	0	0
38	Cirrhosis	0	0	0
39	Cirrhosis	0	0	0
40	Cirrhosis	24	21	19
41	Cirrhosis	0	0	0
42	Cirrhosis	13	10	7
43	Cirrhosis	0	0	0
44	Cirrhosis	0	0	0
45	Normal	0	0	0
46	Normal	0	0	0
47	Normal	0	0	0
48	Normal	0	0	0
49	Normal	0	0	0
50	Normal	0	0	0

Secondly, standard LBP is used and Otsu method is applied on that LBP images. LBP radius (R) is used to compute the LBP operator as shown in Fig. 3. If radius (R) is 1, 2 and 3 then matrix become of 3 × 3, 5 × 5 and 7 × 7 respectively. Then, 3, 4 and 5 connected pixels as explained earlier and are getting these all counts for all three radiuses separately as shown in Table 2. There is again no specific result as observed from table.

**Table 2:** 3, 4 and 5 connected counts using LBP with Otsu method

Sr. No.	Liver	LBP radius = 1, matrix = 3 × 3			LBP radius = 2, matrix = 5 × 5			LBP radius = 3, matrix = 7 × 7		
		3 connected black objects	4 connected black objects	5 connected black objects	3 connected black objects	4 connected black objects	5 connected black objects	3 connected black objects	4 connected black objects	5 connected black objects
1	Cirrhosis	719	554	419	669	536	426	582	464	363
2	Cirrhosis	624	451	334	556	416	317	520	382	288
3	Cirrhosis	709	542	409	585	451	349	589	469	381
4	Cirrhosis	597	471	369	593	486	396	575	473	393
5	Cirrhosis	597	428	307	572	414	295	491	343	237
6	Cirrhosis	551	387	275	500	357	263	423	267	167
7	Cirrhosis	798	641	515	761	628	518	737	622	526
8	Cirrhosis	713	558	436	682	551	443	626	509	414
9	Cirrhosis	724	566	449	660	520	405	635	499	403
10	Cirrhosis	684	499	369	689	526	409	512	368	271
11	Cirrhosis	594	438	325	567	430	319	536	411	317
12	Cirrhosis	795	659	553	743	618	508	714	589	480
13	Cirrhosis	611	456	345	468	348	252	529	410	323
14	Cirrhosis	757	609	496	796	668	566	768	661	574
15	Cirrhosis	707	555	434	681	559	454	617	511	423
16	Cirrhosis	572	411	304	636	488	382	587	459	359
17	Cirrhosis	582	431	319	581	435	332	582	466	379
18	Cirrhosis	536	358	241	554	401	292	556	407	299
19	Cirrhosis	622	457	337	637	507	401	617	506	414
20	Cirrhosis	814	697	591	746	643	553	709	621	542
21	Cirrhosis	717	564	447	716	582	474	663	542	450
22	Cirrhosis	723	570	439	729	593	478	723	597	484
23	Cirrhosis	813	657	529	783	644	527	726	601	497
24	Cirrhosis	666	498	375	637	482	363	619	482	378
25	Cirrhosis	630	506	405	622	510	419	569	471	388
26	Cirrhosis	652	495	378	619	470	369	607	476	386
27	Cirrhosis	643	486	362	649	517	416	582	475	388
28	Cirrhosis	610	475	369	620	480	376	554	437	347
29	Cirrhosis	747	612	497	719	598	496	696	581	480
30	Cirrhosis	618	469	358	653	528	424	618	507	423
31	Cirrhosis	799	680	587	756	658	570	695	611	537
32	Cirrhosis	579	421	298	583	440	327	589	462	355
33	Cirrhosis	792	635	506	753	609	488	719	596	498
34	Cirrhosis	674	527	414	670	531	421	657	533	437
35	Cirrhosis	715	582	473	728	603	509	596	502	423
36	Cirrhosis	774	635	517	751	623	515	715	594	495
37	Cirrhosis	748	596	464	765	635	527	731	627	531
38	Cirrhosis	514	367	269	513	377	288	518	404	324
39	Cirrhosis	728	582	456	736	611	506	705	577	474
40	Cirrhosis	608	477	375	630	512	416	569	470	388
41	Cirrhosis	637	474	347	638	485	358	639	501	390
42	Cirrhosis	737	609	510	732	617	526	706	612	529
43	Cirrhosis	708	549	423	675	529	405	642	517	412
44	Cirrhosis	544	407	303	511	378	276	506	386	295
45	Normal	517	343	237	444	297	210	415	279	187
46	Normal	648	464	333	680	516	391	670	531	423
47	Normal	605	428	300	616	444	332	654	511	410
48	Normal	582	391	264	477	318	218	418	282	198
49	Normal	658	472	333	712	541	427	734	592	482
50	Normal	590	391	261	622	450	324	647	489	372

Finally, modified LBP (DLBP) is used and Otsu method is applied on that DLBP images. Then, again 3, 4 and 5 connected pixels are getting these all counts for all three radiuses separately as shown in Table 3. This is giving the specific result for identification of liver cirrhosis from normal liver.

**Table 3:** 3, 4 and 5 Connected Counts using DLBP with Otsu method

Sr. No.	Liver	LBP radius = 1, matrix = 3 × 3			LBP radius = 2, matrix = 5 × 5			LBP radius = 3, matrix = 7 × 7		
		3 connected black objects	4 connected black objects	5 connected black objects	3 connected black objects	4 connected black objects	5 connected black objects	3 connected black objects	4 connected black objects	5 connected black objects
1	Cirrhosis	182	98	50	271	189	127	264	191	136
2	Cirrhosis	153	85	47	235	175	132	218	153	111
3	Cirrhosis	112	74	49	279	202	146	293	213	155
4	Cirrhosis	40	29	21	148	112	92	186	152	122
5	Cirrhosis	208	123	68	189	114	70	165	94	57
6	Cirrhosis	176	90	48	189	109	62	124	58	26
7	Cirrhosis	222	155	110	304	227	171	346	272	217
8	Cirrhosis	154	94	51	233	161	112	232	163	117
9	Cirrhosis	176	107	68	248	184	137	223	155	106
10	Cirrhosis	189	100	56	270	185	127	255	172	114
11	Cirrhosis	212	136	89	255	173	114	270	189	132
12	Cirrhosis	117	77	49	236	177	130	228	164	119
13	Cirrhosis	33	17	10	104	68	44	136	98	71
14	Cirrhosis	121	90	68	237	186	148	245	195	159
15	Cirrhosis	78	51	36	183	131	95	189	136	98
16	Cirrhosis	71	42	27	159	106	77	162	111	79
17	Cirrhosis	90	57	34	165	117	82	159	105	71
18	Cirrhosis	73	38	18	150	85	51	163	98	57
19	Cirrhosis	80	56	40	184	137	105	217	161	125
20	Cirrhosis	113	80	55	232	182	139	292	245	200
21	Cirrhosis	102	60	35	260	183	133	231	170	127
22	Cirrhosis	155	96	56	295	211	138	285	204	142
23	Cirrhosis	156	100	65	310	224	159	263	187	137
24	Cirrhosis	129	76	47	251	170	118	255	172	122
25	Cirrhosis	104	70	45	221	163	118	276	216	170
26	Cirrhosis	132	79	49	259	187	139	243	180	135
27	Cirrhosis	118	76	49	270	209	161	292	229	178
28	Cirrhosis	89	54	32	180	123	85	198	146	110
29	Cirrhosis	141	97	62	293	231	179	294	223	175
30	Cirrhosis	95	71	54	199	146	111	229	171	132
31	Cirrhosis	130	98	74	215	171	137	300	255	222
32	Cirrhosis	103	67	39	224	163	119	252	190	142
33	Cirrhosis	146	95	63	306	228	169	301	223	167
34	Cirrhosis	102	70	49	217	165	125	244	192	154
35	Cirrhosis	151	115	89	297	239	188	290	232	184
36	Cirrhosis	98	59	30	234	174	131	258	197	157
37	Cirrhosis	94	57	31	237	170	120	274	200	145
38	Cirrhosis	117	76	49	242	177	131	241	177	136
39	Cirrhosis	149	93	57	290	209	149	290	218	165
40	Cirrhosis	94	65	44	205	160	129	226	176	145
41	Cirrhosis	100	51	20	211	133	85	267	184	121
42	Cirrhosis	102	80	64	240	199	169	270	227	195
43	Cirrhosis	81	51	28	201	138	96	232	154	107
44	Cirrhosis	105	68	45	193	137	105	186	127	87
45	Normal	119	55	28	209	127	78	177	100	54
46	Normal	51	24	12	114	68	47	129	79	53
47	Normal	33	15	5	79	43	25	81	44	28
48	Normal	58	22	8	119	63	36	117	62	32
49	Normal	58	28	13	116	68	37	139	92	65
50	Normal	37	16	8	107	54	29	127	75	39

As observed from the Table 3, if the counts of 3 connected pixels for radius 1 are <58 then the liver is normal liver and if the counts are >71 then the liver is cirrhotic liver. Similarly, the counts for 3, 4 and 5 connected pixels for radius 1, 2 and 3 are explained in Table 4 for normal liver and cirrhotic liver.

**Table 4:** 3, 4 and 5 connected counts for normal liver and cirrhotic liver using DLBP with Otsu method

Counts	Radius = 1		Radius = 2		Radius = 3	
	For normal liver	For cirrhotic liver	For normal liver	For cirrhotic liver	For normal liver	For cirrhotic liver
Counts of 3 connected pixels	0–58	Above from 71	0–119	Above from 148	0–139	Above from 159
Counts of 4 connected pixels	0–28	Above from 42	0–68	Above from 106	0–92	Above from 111
Counts of 5 connected pixels	0–13	Above from 27	0–47	Above from 62	0–65	Above from 79

There are some problem in counts for cirrhotic liver because which are assuming as cirrhotic liver that might be at the first stage of cirrhosis i.e. fibrosis. So, further methodology can be amending to stage the cirrhosis. That’s why radius 2 and radius 3 is chosen. As the granular and scarring increases that much part is cirrhosis. If it does not lie in the range of cirrhosis then it might be fibrosis.

For statistical interpretation, widely accepted classifiers are used. Here, two classifiers have been selected; one is support vector machine (SVM) and other one is K-nearest neighbor (KNN) support vector machine (SVM) has been chosen for the classification task because classifier designs which use regularization like SVM are less prone to over fitting and obtain good generalization performance to a certain extent even without feature space dimensionality reduction. KNN has been chosen because KNN is non-parametric, i.e. it makes no assumption about the data distribution. Accuracy of both the classifier will justify our methodology and decision.

**Fig. 6:**
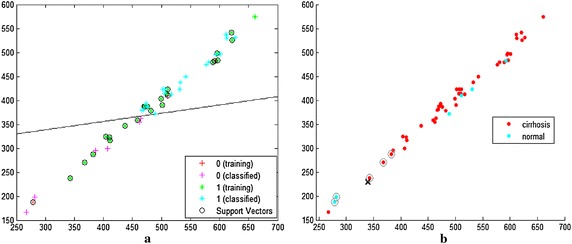
**a** SVM classification of OTSU with LBP method, **b** KNN classification of OTSU with LBP method

**Fig. 7:**
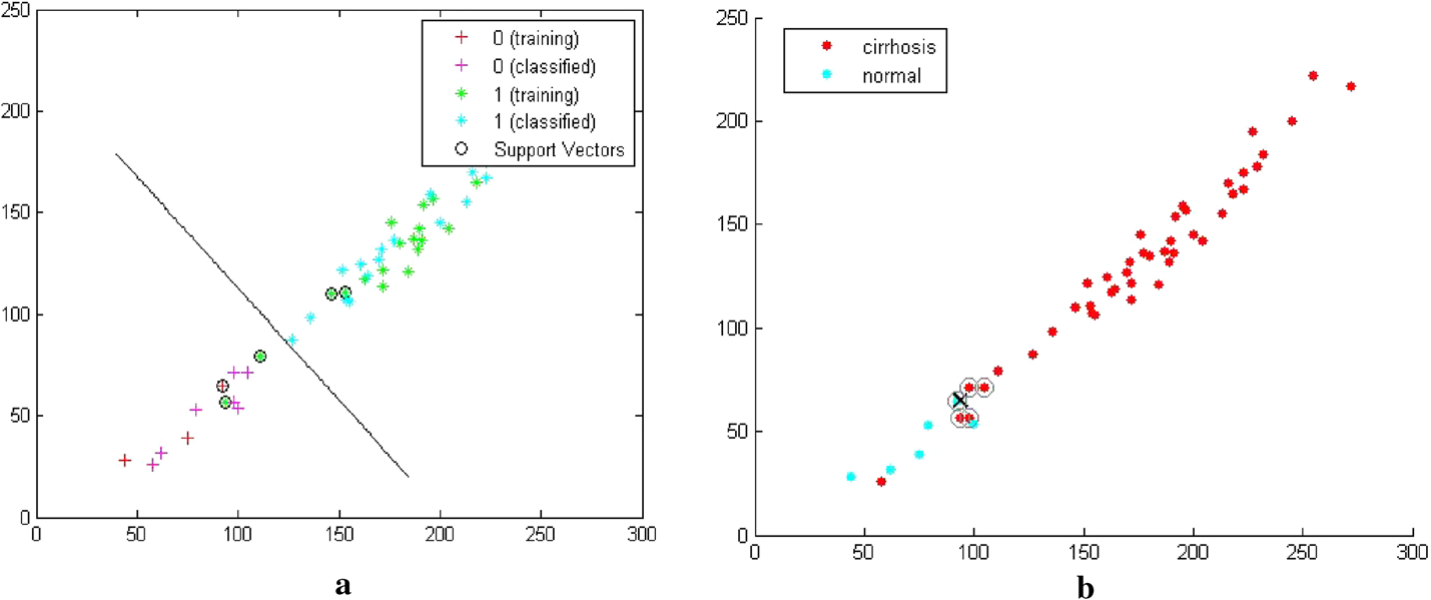
**a** SVM classification of OTSU with DLBP method, **b** KNN classification of OTSU with DLBP method

Figures 6 and 7 are the classifications of OTSU with LBP method and OTSU with DLBP method respectively. In these classifiers, x axis is 4 connected black objects and y axis is 5 connected black objects for LBP radius of 3.

In SVM classification of Figs. 6a and 7a, pink plus sign ‘+’ indicates the normal liver and blue plus sign ‘+’ indicates the cirrhotic liver. The line divides the region of the normal and cirrhotic liver. When OTSU with LBP method is used, there are only 32 out of 44 blue plus signs falls in the cirrhosis region and 5 out of 6 red plus signs in normal region as shown in Fig. 6a. So accuracy is 72.7 % for the cirrhotic liver and 83.3 % for normal liver of OTSU with LBP method. When OTSU with DLBP is used, there are 42 out of 44 blue plus signs falls in the cirrhosis region and all red plus signs in normal region as shown in Fig. 7a. So accuracy is 95.45 % for the cirrhotic liver and 100 % for normal liver of OTSU with DLBP method.

In KNN classification of Figs. 6b and 7b, blue dots for normal liver and red dots for cirrhotic liver. In this, all blue dots are accumulated together and red dots are accumulated together. In this classification, plus (+) sign is the point that separates the region of cirrhosis and normal liver and circles are the nearest neighbors of this plus (+) sign. When OTSU with LBP method is used, there are 40 out of 44 red dots falls in the cirrhosis region and 2 out of 6 blue dots falls in the normal region as shown in Fig. 6(b). So, accuracy is 90.9 % for the cirrhotic liver and 33.33 % for the normal liver of OTSU with LBP method. When OTSU with DLBP method is used, there are 43 out of 44 red dots falls in the cirrhosis region and 5 out of 6 red dots falls in the normal region as shown in Fig. 7b. So, accuracy is 97.72 % for the cirrhotic liver and 83.33 % for the normal liver of OTSU with DLBP method. The sensitivity is 97.75 and specificity is 83.3 % of this method.

**Table:** 

Methods	Recognition rate	
	Using SVM classifier (%)	Using KNN classifier (%)
OTSU with LBP method	72.7	90.9
OTSU with DLBP method	95.45	97.72

## Conclusions

In this study, a modified LBP with OTSU method has been proposed for better identification of texture in ultrasound images of liver in which uncertainty is introduced by inherent noise. The proposed inspection technique was evaluated on a real dataset of 44 templates of 44 cirrhotic liver and 6 templates of 6 normal liver ultrasound images. The promising results achieved in detecting cirrhotic liver. SVM classifier has given the accuracy of 95.45 % for cirrhotic liver and 100 % for normal liver. Similarly, KNN has given the accuracy of 97.72 % for cirrhotic liver and 83.3 % for normal liver. The sensitivity is 97.2 % and specificity is 83.3 %. In the future study; the performance of this technique can be investigated on ultrasound images to classify different stages of cirrhotic liver.
